# Surgery, Octreotide, Temozolomide, Bevacizumab, Radiotherapy, and Pegvisomant Treatment of an *AIP* Mutation‒Positive Child

**DOI:** 10.1210/jc.2019-00432

**Published:** 2019-05-24

**Authors:** Pinaki Dutta, Kavita S Reddy, Ashutosh Rai, Anil K Madugundu, Hitendra S Solanki, Anil Bhansali, Bishan D Radotra, Narendra Kumar, David Collier, Donato Iacovazzo, Prakamya Gupta, Remya Raja, Harsha Gowda, Akhilesh Pandey, Jagtar Singh Devgun, Márta Korbonits

**Affiliations:** 1 Department of Endocrinology, Postgraduate Institution of Medical Education and Research, Chandigarh, India; 2 Institute of Bioinformatics, International Tech Park, Bangalore, Karnataka, India; 3 Department of Translational and Regenerative Medicine, Postgraduate Institution of Medical Education and Research, Chandigarh, India; 4 Institute of Genetic Medicine, Division of Proteomics, Mayo Clinic, Rochester, Minnesota; 5 Manipal Academy of Higher Education, Manipal, Karnataka, India; 6 Center for Individualized Medicine and Department of Laboratory Medicine and Pathology, Mayo Clinic, Rochester, Minnesota; 7 School of Biotechnology, KIIT University, Bhubaneswar, India; 8 Department of Histopathology, Postgraduate Institution of Medical Education and Research, Chandigarh, India; 9 Department of Radiotherapy, Postgraduate Institution of Medical Education and Research, Chandigarh, India; 10 Centre for Endocrinology, William Harvey Research Institute, Barts and The London School of Medicine, Queen Mary University of London, London, United Kingdom; 11 Indian Council of Medical Research, New Delhi, India; 12 Department of Pathology, Maharishi Markandeshwar Institute of Medical Science and Research, Ambala, Haryana, India

## Abstract

**Context:**

Inactivating germline mutations in the aryl hydrocarbon receptor interacting protein (*AIP*) gene are linked to pituitary adenoma predisposition. Here, we present the youngest known patient with *AIP*-related pituitary adenoma.

**Case Description:**

The patient presented at the age of 4 years with pituitary apoplexy and left ptosis with severe visual loss following a 1-year history of abdominal pain, headaches, and rapid growth. His IGF-1 level was 5× the upper limit of normal, and his random GH level was 1200 ng/mL. MRI showed a 43 × 24 × 35‒mm adenoma with suprasellar extension invading the left cavernous sinus (Knosp grade 4). After transsphenoidal surgery, histology showed a grade 2A sparsely granulated somatotropinoma with negative O6-methylguanine-DNA methyltransferase and positive vascular endothelial growth factor staining. Genetic testing identified a heterozygous germline nonsense *AIP* mutation (p.Arg81Ter). Exome sequencing of the tumor revealed that it had lost the entire maternal chromosome-11, rendering it hemizygous for chromosome-11 and therefore lacking functional copies of *AIP* in the tumor. He was started on octreotide, but because the tumor rapidly regrew and IGF-1 levels were unchanged, temozolomide was initiated, and intensity-modulated radiotherapy was administered 5 months after surgery. Two months later, bevacizumab was added, resulting in excellent tumor response. Although these treatments stabilized tumor growth over 4 years, IGF-1 was normalized only after pegvisomant treatment, although access to this medication was intermittent. At 3.5 years of follow-up, gamma knife treatment was administered, and pegvisomant dose increase was indicated.

**Conclusion:**

Multimodal treatment with surgery, long-acting octreotide, radiotherapy, temozolomide, bevacizumab, and pegvisomant can control genetically driven, aggressive, childhood-onset somatotropinomas.

## Teaser

AIP mutation-driven gigantism with pituitary apoplexy and aggressive tumor growth was controlled by multimodal treatment: surgery, octreotide-LAR, radiotherapy, temozolomide, bevacizumab, and pegvisomant.

Screening for genetic alterations is recommended in patients with childhood-onset pituitary adenomas even with no apparent family history (1, 2). Here, we report on a 4-year-old child who presented with an aggressive somatotropinoma harboring a heterozygous aryl hydrocarbon receptor interacting protein (*AIP)* mutation and who, in addition to surgery and radiotherapy, required a somatostatin analogue, pegvisomant, temozolomide, and bevacizumab therapy to control tumor growth and hormone excess. Written informed consent was obtained from the parents of the patient for publication of this case report and associated images.

## Case Presentation

**Figure 1.**
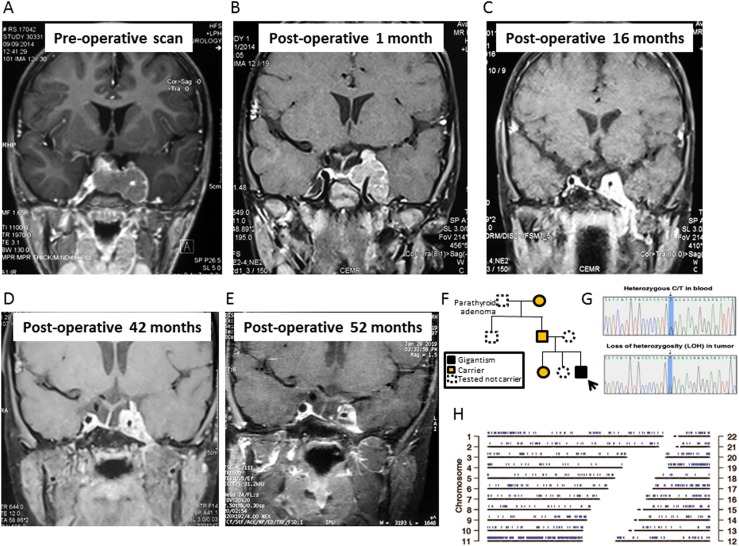
Coronal T1-weighted MRI of the sella showing a large pituitary adenoma with left cavernous sinus invasion (A) at diagnosis; (B) 1 month after surgery; (C) 16 months after surgery; (D) 42 months after surgery, just before gamma knife therapy; and (E) 52 months after surgery, 9 months after gamma knife therapy. (F) The family pedigree chart shows incomplete penetrance. The arrow marks the proband. (G) Sanger sequencing shows a heterozygous *AIP* c.241C>T mutation in blood leukocytes and loss of heterozygosity in the tumor. (H) Genomic positions with loss of heterozygosity in the tumor sample were identified using VarScan and are marked in blue.

A 4-year-old boy presented to the neurosurgical department with intense headaches, vomiting, ptosis, and only light perception in the left eye following a 12-month history of abdominal pain and headaches. Acanthosis nigricans, multiple café-au-lait spots, excessive sweating, and dental malocclusion were noted. A clinical diagnosis of pituitary apoplexy was made. He had tall stature [height +2.5 SD score (SDS); midparental height +0.3 SDS] developing over the previous year (growth velocity, 12.8 cm/y; 50th percentile = 7 cm/y). The child was irritable, aggressive, and hyperkinetic with poor attention span. His serum IGF-1 level was 5× the upper limit of normal (ULN) and his GH level was 1200 ng/mL, whereas his prolactin, free T4, TSH, 9 am cortisol, hemoglobin A1c, calcium, and electrolyte levels were within the normal range. MRI showed a 43 × 24 × 35‒mm tumor with suprasellar and left cavernous extension (Knosp grade 4) and evidence of acute bleeding in the tumor (Fig. 1A). Craniospinal MRI, whole body gallium-68 DOTATATE scan, and cerebrospinal fluid examination for tumor cells excluded systemic dissemination of the disease. There was no known family history of pituitary adenoma or gigantism (Fig. 1F), and family members were well. His paternal grandfather had a history of hyperparathyroidism due to a parathyroid adenoma. Genetic analysis revealed a previously repeatedly described (3–6) germline nonsense *AIP* mutation (c.241C>T; p.Arg81Ter) (Fig. 1G) in the proband and three family members with normal clinical, biochemical, and MRI assessments. Whole-exome sequencing on peripheral and tumor DNA revealed that 1293 variants of the 1311 detected heterozygous germline chromosome 11 variants were hemizygous (suggesting loss of heterozygosity) in tumor DNA, strongly suggesting loss of the entire maternal chromosome 11 (Fig. 1G and 1H). Somatic mutations in the tumor were analyzed using VarScan (v2.6.x) and MuTect (v1.1.4), whereas germline sequence variations were called using GATK’s UnifiedGenotyper. The effect of variants on protein function was predicted using SIFT and PolyPhen2 scores, and annotation was performed using ANNOVAR (release: March 2015) and SnpEff (v4.1). OMIM, ClinVar, and COSMIC databases were used for annotation of identified variants. Minor allele frequencies were obtained from the dbSNP (build 142), 1000 Genomes, and ExAC databases.

## Treatment

**Figure 2.**
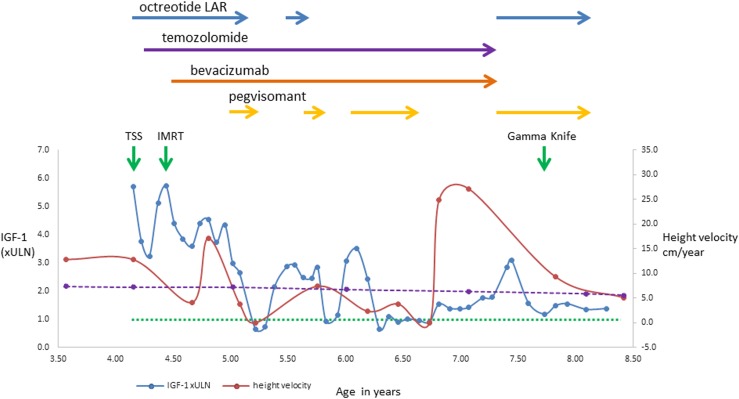
IGF-1 and height velocity during multimodal therapy. IGF-1 is expressed as times ULN (×ULN). The dotted green line represents the ULN for IGF-1, and the dashed purple line represents the 50th percentile height velocity for ethnically matched boys (7). IMRT, fractionated intensity-modulated radiotherapy; TSS, transsphenoidal surgery.

The patient underwent transsphenoidal surgery after diagnosis. Histopathology showed a sparsely granulated somatotropinoma with areas of hemorrhage, suggestive of recent apoplexy. The Ki-67 index was 12%, with negative p53, moderately positive vascular endothelial growth factor (VEGF) staining, negative O6-methylguanine-DNA methyltransferase (MGMT) immunostaining, and methylation of the MGMT promoter by PCR. The tumor could be classified as grade 2A (Trouillas classification). Immediate postoperative MRI showed a large left parasellar remnant. Octreotide-long-acting release (LAR) was implemented (20 mg per month) without effect on IGF-1 levels (Fig. 2). One month after surgery, there was a substantial increase in the size of the parasellar tumor remnant (Fig. 1B); therefore, the patient was started on temozolomide (180 mg/d, 5 d/mo) (Fig. 2). Four months after surgery, fractionated intensity-modulated radiotherapy (40 Gy; 23 sessions) was given to the left cavernous sinus remnant and IV anti-VEGF bevacizumab was initiated (first cycle: 5 mg/kg; second to fifth cycles: two weekly cycles, 7.5 mg/kg; from cycle 6 onward: 10 mg/kg). Postoperative MRIs at 3- to 6-month intervals showed a steady reduction in tumor size (Fig. 1C).

By the sixth postoperative month, his left ptosis had resolved, but there was no improvement in the visual acuity of the left eye. Serum GH (>50 ng/mL) and IGF-1 (3× ULN) levels remained high, whereas the tumor showed significant shrinkage. One year after surgery, pegvisomant was added at the dose of 10 mg/d, and IGF-1 fell to a normal level (0.6× ULN). Octreotide was stopped, but temozolomide and bevacizumab were continued. The patient’s IGF-1 level remained in the normal range for the following 2 months. For financial reasons, the pegvisomant supply was unreliable, and the drug dose had to be first reduced and then stopped; however, it was later intermittently reinstated (Fig. 2). When pegvisomant was administered, the IGF-1 level rapidly dropped and remained stable, whereas it was elevated when pegvisomant was not administered (Fig. 2).

Four years after surgery, both temozolomide (38 months of treatment) and bevacizumab (35 months of treatment) were stopped because no further tumor shrinkage was seen, to reduce toxicity, and given that pubertal induction would be initiated soon and temozolomide is an alkylating agent with potential gonadal toxicity. Octreotide-LAR (20 mg/mo) and pegvisomant (10 mg/d) were restarted, as there are data suggesting synergism between these two medications (8). After a multidisciplinary team discussion and considering the persistently uncontrolled GH excess at 42 months after surgery, gamma knife radiotherapy was administered to the tumor remnant (13 Gy at 50%) (Fig. 1D).

The patient’s current height is 145 cm (99.5th percentile; SDS +2.58). The latest MRI showed a stable tumor remnant with some necrosis (Fig. 1E). The patient is receiving T4 and hydrocortisone replacement, and pubertal initiation is planned in a few years; careful timing of this may help to optimize final height. Current treatment includes monthly 20-mg octreotide-LAR and daily 10-mg pegvisomant, with IGF-1 ranging from 1.2× to 1.4× ULN. Because of the high growth velocity and suboptimal IGF-1 control, pegvisomant dose increase is indicated.

## Discussion

*AIP* mutation‒positive somatotropinomas are often aggressive and treatment resistant, and the onset of first symptoms typically occurs in the second decade of life. This patient, who already had increased growth rate from the age of 3 years, is the youngest known patient with an *AIP* mutation‒positive pituitary somatotropinoma. He is also the first pediatric patient with a pituitary adenoma treated with bevacizumab and the first patient with *AIP* mutation positivity treated with the combination of temozolomide and bevacizumab. The combination of surgery, radiotherapy, octreotide, temozolomide, and bevacizumab controlled his tumor growth, whereas biochemical control was achieved only when pegvisomant was added.

There is now considerable experience with temozolomide in pituitary adenomas in adults (9). Four pediatric patients treated with temozolomide for a pituitary tumor (10–12) were previously described, whereas reports of two more patients with childhood-onset disease and temozolomide treatment in adulthood (13, 14) were published. Temozolomide was initiated in the current case because of aggressive tumor behavior showing rapid enlargement after surgery, with the aim of controlling tumor growth and sensitizing it to radiotherapy (15). Bevacizumab was considered because of positive VEGF immunostaining in the tumor and in view of previous successful use in a patient with a pituitary carcinoma that resulted in prolonged (>8 years) control of tumor growth and survival [(16) and personal communication with Dr Luis Syro, Medellin, Colombia]. VEGF staining is present in somatotroph adenomas, and we have not seen a difference between *AIP* mutation‒positive and *AIP* mutation‒negative cases (17). Both treatments were started with the hope of achieving additive or augmented effects, as data from patients with glioblastoma multiforme showed the combination of these two drugs causing remarkable tumor reduction along with survival benefits [(18) and ClinicalTrials.gov Identifier: NCT00612339, phase II, 2008]. Recently, temozolomide and bevacizumab treatment was stopped, as no further benefit was seen.

Our patient was 3 years old at the time of his first symptoms. Because of the presence of café-au-lait spots, age <5 years at onset, and apparent sporadic presentation, McCune-Albright syndrome and X-linked acrogigantism were considered as differential diagnoses. Technetium-^99^m methylene diphosphonate bone scan and genetic analysis of the tumor tissue ruled out McCune-Albright syndrome. X-linked acrogigantism due to *GPR101* duplication was ruled out with droplet digital PCR analysis (19). MEN1 syndrome was considered because of the paternal grandfather’s parathyroid disease; however, his age at onset of hyperparathyroidism was quite late, and no other MEN1-related manifestations were present in the family. In addition, childhood-onset somatotropinomas are extremely rare in the setting of MEN1 syndrome, in which pediatric GH excess is more likely to arise from GHRH-secreting neuroendocrine tumors. Exome sequencing did not identify pathogenic variants in the *MEN1* gene. The patient was investigated for possible metastatic disease. Although pituitary carcinomas have not been reported in patients with *AIP* mutations, they have been observed in other genetic pituitary adenoma predisposition syndromes [*MEN1* (20, 21), *SDHB* (22), *AIP* mutation‒negative familial isolated pituitary adenoma (23, 24)] and in one patient with Lynch syndrome (25). A literature search identified three cases of pituitary carcinomas in patients with childhood-onset pituitary tumors, although none of them were somatotropinomas (13, 26, 27).

Apoplexy has been associated with *AIP* mutation‒positive tumors (6, 28); currently, it is unclear whether the rapid tumor growth, the younger age, or a specific AIP-related molecular mechanism is responsible for this observation. The apparent sporadic presentation of our patient highlights the low penetrance seen among *AIP* mutation carriers. Loss of the entire maternal (wild-type) copy of chromosome 11 may play a role in his particularly early and aggressive manifestation. The patient showed fluctuations in his growth patterns. Some could be explained by changes in IGF-1 levels, but not all. There was a deceleration in height velocity during the first 6 months after surgery, followed by acceleration again. He also showed rapid growth after temporary cessation of pegvisomant. Growth can be a periodic phenomenon influenced by many factors, including intermittent chondrocyte recruitment and senescence. This programmed senescence does not appear to be caused by hormonal or other systemic mechanisms but is intrinsic to the growth plate itself (7, 29).

In conclusion, patients with somatotropinomas often require multimodal treatment. Although we were able to control aggressive tumor growth with surgery, radiotherapy, temozolomide, bevacizumab, and octreotide-LAR in this patient, excess hormone release was curbed only by pegvisomant. The application of an extensive armamentarium of interventions can control aggressive disease, although costs can be prohibitive even in developed countries, let alone in low-income states.

## Abbreviations:

*AIP*: aryl hydrocarbon receptor interacting protein
octreotide-LAR: octrotide long-acting release
SDS: SD score
ULN: upper limit of normal
VEGF: vascular endothelial growth factor
